# The Action of Different Proportions of Chemical Agents upon the Germs of Emphysema Infectiosum

**Published:** 1884-10

**Authors:** 


					﻿The Action of Different Proportions of Chemical Agents
upon the Germs of Emphysema Infectiosum. Arloing, Cor-
venin and Thomas. Recueil d. Med. Vet., May 15,1882.*
The following have no destroying action upon fresh material:
Alcohol, 90 per cent. Saturated solutions of camphor. Satu-
rated alcoholic solutions of carbolic acid. Glycerine. Ammo-
niac. Acetate of ammonia. Sulphate of ammonia. Borax, 1.5
per cent. Sodium subsulfuricum, 1.5 per cent. Benzin. Sat-
urated preparations of chlorate of soda. Crude lime and wa-
tery preparations of the same. Sulphate of calcium. Magnesium
chloratum, Ferrum sulphuricum, Chinicum sulph., Tannin, of
each 1.5 per cent. Sulphurous acid. Chloroform.
The following had no action upon desiccated material of this
disease :
Oxalic acid. Hypermanganate of potash. Soda. Chlorine.
Sulphate of carbon.
Fresh material was rendered inactive by:
Carbolic acid, one to fifty. Salicylic acid, one to a thousand.
Acidum biboracicum, one to five. Acid sulph., one to twenty.
Acid nit., one to twenty. Acid muriat., one to twenty. Oxalic
acid, saturated solutions of Salicylic acid, Soda caust., one to
five. Potash, saturated solutions, Iodine, Salicylate of Natron,
one to five. Potass, hypermang., one to twenty. Sulphas Cupri,
one to five. Argent, nit., one to a thousand. Corr, sublimat.,
one to five thousand. Bromine, Chlorine.
Dried material is destroyed by:
Carbolic acid, one to fifty. Salicylic acid, one to a thousand.
Argent, nit., one to a thousand. Cupri sulph., one to five. Mu-
riatic acid, one to two. Boracic acid, one to five. Corr, subli-
mate, one to five thousand.
*From the Deutsche Zeitschrift fur Thiermedicin, Vol. 10, Part I.
				

